# SPA-DETR: An Enhanced RT-DETR with Spatial-Preserving Attention and Adaptive Loss for UAV Spectrogram Signal Detection

**DOI:** 10.3390/s26154846

**Published:** 2026-08-01

**Authors:** Conghao Fu, Lu Xu, Yijia Zhang

**Affiliations:** School of Information Science and Engineering, Zhejiang Sci-Tech University, Hangzhou 310018, China; fu_conghao@163.com (C.F.); waiting@zstu.edu.cn (Y.Z.)

**Keywords:** unmanned aerial vehicle (UAV), radio frequency (RF) spectrogram, object detection, RT-DETR, spatial-preserving attention, focal loss

## Abstract

Rapid detection of unauthorized unmanned aerial vehicles (UAVs) via radio frequency (RF) spectrograms is critical for low-altitude security. However, standard object detectors struggle to locate transient, frequency-hopping UAV signals because their microscopic spatial footprints are easily discarded by conventional lossy downsampling and overwhelmed by complex background noise. To overcome this limitation, we propose SPA-DETR, a custom architecture based on the RT-DETR framework. The core of our design is the Spatial-Preserving Attention (SPA) block, which integrates Space-to-Depth Convolution (SPDConv) with a Parallel Patch-Aware Attention (PPA) module. By replacing traditional pooling mechanisms, the SPA block preserves the spatial details of weak signals without information loss, while the PPA module concurrently filters out ambient background interference. Furthermore, to address the severe foreground–background imbalance in RF spectrograms, we introduce an Adaptive Threshold Focal Loss (ATFL). Operating exclusively during training, ATFL prevents background noise gradients from dominating the learning process, forcing the network to focus on hard-to-detect signal patches without adding computational overhead during inference. Experiments on our public RFUAV dataset validate the approach. SPA-DETR achieves an $mAP_{50:95}$ of 86.8% and an $AP_{S}$ of 85.6%, improving upon the baseline RT-DETR-R18 by 4.9% and 5.2%, respectively. Operating at 235.2 FPS with only 23.74 M parameters, SPA-DETR outperforms contemporary detectors such as YOLOv10m, as well as heavier models like YOLOv8m and RT-DETR-R50, highlighting its efficiency and practical value for real-time low-altitude security applications.

## 1. Introduction

The rapid development of unmanned aerial vehicle (UAV) technology has transformed civilian logistics and modern electronic warfare. However, their widespread use has increased the risk of “low–slow–small” aerial threats, making unauthorized incursions a persistent concern for low-altitude security [1,2]. Traditional radar systems often struggle to distinguish small UAVs from birds or urban obstacles [3]. Passive radio frequency (RF) sensing provides a practical alternative by transforming intercepted time-domain signals into 2D spectrograms [4]. This enables the use of computer vision (CV) algorithms to analyze the temporal-frequency patterns of UAV communication links. Nonetheless, the sparse and transient nature of frequency-hopping signals requires detection systems with high sensitivity and real-time processing capabilities.

While mainstream object detection frameworks, such as the YOLO series and DETR variants, perform well on natural images, applying them directly to RF spectrograms yields suboptimal results. The main issue is the small physical footprint of frequency-hopping signals. During their brief dwell time, these pulses occupy very few pixels. Standard architectures extract semantic features through successive downsampling stages, such as max-pooling or strided convolutions. This spatial compression benefits typical large objects, but it acts as a lossy filter for weak UAV signals, diluting critical spatial details before deeper layers can process them [5]. Moreover, RF spectrograms suffer from a severe foreground–background imbalance, with ambient noise dominating the image. This causes a high false-alarm rate, as standard loss functions struggle to prioritize rare signal pulses over background clutter [6].

To address these structural issues, we propose SPA-DETR, a custom architecture based on the Real-Time Detection Transformer (RT-DETR) [7] for weak UAV signal detection. To preserve the spatial footprint of hopping signals, we fundamentally redesign the network’s downsampling paradigm. Rather than relying on conventional lossy operations, we introduce a Spatial-Preserving Attention (SPA) block, which integrates Space-to-Depth Convolution (SPDConv) with a Parallel Patch-Aware Attention (PPA) module. We deploy this block across both the early backbone and the multi-scale fusion neck. The SPDConv operation retains the geometric details of transient signals without spatial loss, while the subsequent PPA mechanism filters out the accompanying background noise. In addition to these structural changes, we introduce an Adaptive Threshold Focal Loss (ATFL) to handle the extreme sample disparity. This objective function dynamically shifts the learning focus toward hard-to-detect signal patches. This direct approach reduces false alarms without adding computational overhead during inference.

The primary contributions of this work are summarized as follows:

(1) We design the SPA block to replace conventional downsampling. By combining the lossless dimension reduction of SPDConv with the noise-filtering of PPA across the backbone and neck stages, this module maintains the spatial integrity of weak frequency-hopping signals and isolates them from ambient interference.
(2) We propose the ATFL to address the severe foreground–background imbalance in RF spectrograms. It dynamically adjusts the optimization focus based on target confidence, improving the network’s sensitivity to small targets in noisy environments.
(3) We conduct extensive experiments on the public RFUAV dataset. With a compact size of 23.74 M parameters, SPA-DETR achieves an $mAP_{50:95}$ of 86.8% and an $AP_{S}$ of 85.6% at 235.2 FPS. It consistently outperforms contemporary detectors like YOLOv8m, YOLOv10m, and the RT-DETR-R50 baseline.

## 2. Related Work

RF-based UAV detection has transitioned from manual feature engineering to data-driven deep learning methodologies. Early approaches primarily relied on statistical signal processing to identify UAV signatures. For instance, energy detection serves as a foundational technique by evaluating received signal power against predefined thresholds. Recently, Sova et al. proposed a burst-aware cascade framework that leverages low-cost energy detection as a preliminary filter [8]. Similarly, Magiera introduced a non-parametric lightweight classification paradigm based on the physical statistical characteristics of RF transmissions, extracting features such as bandwidth and pulse duration [9]. However, while computationally efficient, these threshold-based models remain highly susceptible to fluctuations in low signal-to-noise ratio (SNR) environments, often suffering from severe false-alarm rates when facing channel multipath fading. To improve noise resilience, researchers have extensively investigated cyclostationary feature analysis and RF fingerprinting, which exploit the inherent periodicity and transient behaviors of modulated signals. For example, Morge-Rollet et al. explored physical-layer protocol statistical fingerprints (PLPSF), utilizing power spectral entropy and packet time-sequence features to distinguish specific UAV models [10]. Furthermore, Ezuma et al. transformed 1D time-domain signals into energy–time–frequency trajectories to extract transient energy features for high-precision classification [11]. Despite demonstrating superior robustness against ambient interference, these methods demand complex mathematical transformations (e.g., cyclic autocorrelation), incurring significant computational overhead that inherently restricts their viability for real-time deployment [12].

To bypass the limitations of handcrafted features and computational bottlenecks, research shifted towards transforming 1D time-series data into 2D time–frequency spectrograms, enabling the direct application of deep learning. Initially, researchers employed standard CNN-based classifiers to categorize the entire spectrogram. For instance, Xin et al. introduced a Complex-Valued CNN (CV-CNN) that jointly processes power spectral density and Sobel edge features to significantly enhance anti-interference capabilities in low SNR regimes [13]. While effective for simple presence detection or global classification, these image-level classifiers inherently lack spatial awareness. They fail to precisely localize the exact time and frequency boundaries of multiple transient pulses within heavily cluttered backgrounds. Consequently, recent studies have adapted mainstream object detectors to not only classify but also physically bound UAV signals within the spectrogram. For example, Zhu et al. designed a high-resolution passive system that utilizes the YOLO framework to explicitly anchor frequency-hopping signal regions before applying a secondary CNN classification. Nevertheless, directly applying these standard bounding-box architectures to highly transient frequency-hopping signals remains suboptimal. Unlike continuous communication links that form solid shapes, frequency-hopping pulses appear as isolated, microscopic energy patches with minimal spatial footprints. Conventional convolutional architectures rely on lossy downsampling, which inadvertently discards these discrete footprints amid background clutter, explicitly requiring the targeted structural modifications we propose.

Detecting small objects remains a fundamental challenge in computer vision. Under standard metrics like MS COCO, targets smaller than 32×32 pixels lose critical information during the successive downsampling stages of conventional Convolutional Neural Networks (CNNs). To address this spatial degradation, SPDConv [14] was introduced to achieve lossless downsampling by reorganizing spatial dimensions into the channel axis. Recent architectures have successfully applied this concept; for example, DBYOLOv8 utilizes SPDConv for complex drone imagery [15], and SPT-YOLO applies it to infrared small target detection to preserve fine-grained geometric features [16]. However, retaining all spatial information also preserves the background noise inherent in complex environments. Consequently, researchers often combine spatial preservation techniques with attention mechanisms. For the extreme sparsity of frequency-hopping signals, standard attention modules are insufficient. A more aggressive, patch-aware filtering approach is necessary to suppress ambient clutter without attenuating the weak signal.

Transformer-based architectures, such as DETR [17], have simplified object detection by framing it as an end-to-end set prediction problem. This eliminates post-processing steps like Non-Maximum Suppression (NMS), which are required by YOLO-family models and introduce variable inference latency. Building on this, RT-DETR bridged the gap between Transformer accuracy and CNN inference speeds. Nevertheless, the baseline RT-DETR relies heavily on deep semantic features from the backbone, often neglecting the low-level textural details necessary for small-object localization. Recent adaptations for aerial imagery, including SO-RTDETR [18] and SDU-RTDETR [19], address this by enhancing multi-scale feature fusion and utilizing shallower detection layers (e.g., the P2 layer). While these enhancements improve spatial awareness, they increase computational overhead and are not optimized for the severe class imbalance of RF spectrograms. Our proposed SPA-DETR addresses this gap by unifying the SPA block and ATFL. This architecture achieves a balance between sensitivity to micro-signals and real-time efficiency without significantly increasing the parameter count.

## 3. Proposed Method

### 3.1. Overall Architecture of SPA-DETR

**Figure 1:**
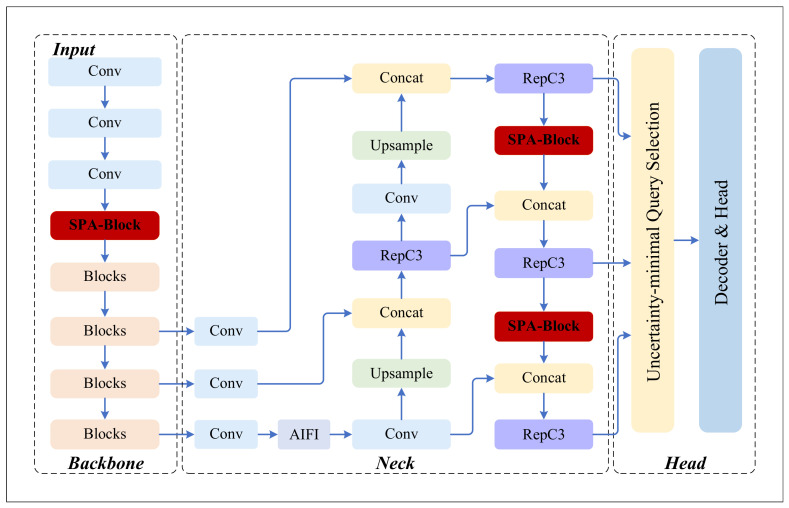
Overall architecture of the proposed SPA-DETR. The SPA blocks are strategically integrated into the early stage of the ResNet-18 backbone and the bottom-up pathways of the hybrid encoder to prevent the loss of microscopic signal footprints.

To address the challenges of detecting weak frequency-hopping signals, we introduce SPA-DETR, a custom architecture based on the RT-DETR-R18 framework, as illustrated in Figure 1. While it retains the standard three-part structure—a feature extraction backbone, a multi-scale hybrid encoder, and a Transformer-based decoder—it fundamentally changes how spatial information is processed.

The input spectrogram first passes through the ResNet-18 backbone [20]. However, instead of using the conventional max-pooling operation at the early P2 stage, SPA-DETR integrates our proposed SPA block. This replacement prevents the loss of the tiny geometric footprints of UAV signals during the initial stages of feature extraction. After this initial extraction, the hierarchical features enter the hybrid encoder for multi-scale fusion. Here, we embed additional SPA blocks within the bottom-up pathways of the feature pyramid, replacing the standard strided convolutions typically used for downsampling. This cross-stage design ensures that the fine spatial details preserved in the early layers are successfully propagated to higher levels without accumulating background noise. Finally, the decoder uses these filtered, multi-scale feature maps for bounding box regression and classification.

During training, the entire network is optimized using the ATFL. This loss function dynamically adjusts the learning gradients, forcing the network to prioritize rare signal patches over the dominant background clutter.

### 3.2. SPA-Block

**Figure 2:**
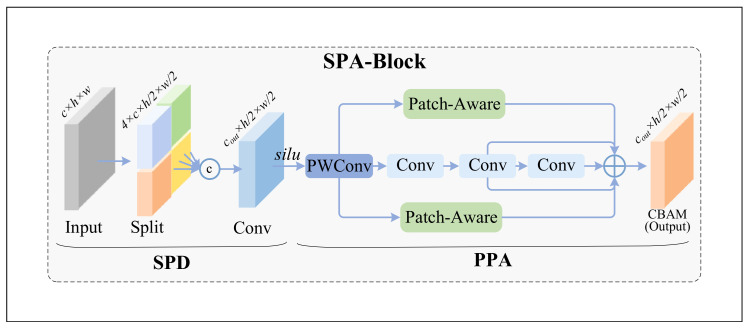
Detailed internal structure of the SPA-block, comprising the SPD for spatial restructuring and the PPA module for feature purification.

To circumvent the information loss dictated by conventional spatial compression, we engineer the SPA, the detailed internal structure of which is depicted in Figure 2. In standard convolutional architectures, downsampling operations—such as max-pooling or strided convolutions—achieve an expanded receptive field by discarding intermediate pixels. For microscopic frequency-hopping signals that span only a minimal pixel region, such aggressive decimation often results in the complete erasure of the target’s geometric footprint.

Addressing this structural vulnerability, the primary phase of our SPA-block adopts SPDConv. Rather than discarding spatial data, SPDConv downsizes the feature map by physically reorganizing spatial blocks into the channel dimension. Given an intermediate feature map $X\in R^{C\times H\times W}$, a scaling factor of s=2 partitions the spatial dimensions into sub-tensors. These sub-tensors are subsequently concatenated along the channel axis to yield a newly structured tensor $X^{\prime }\in R^{4C\times \frac{H}{2}\times \frac{W}{2}}$, followed by a non-strided convolution to regulate the channel depth. This lossless transformation guarantees that every raw pixel of the weak UAV signal is explicitly retained.

However, the indiscriminate preservation of spatial data introduces a critical secondary challenge: the complex background noise, which dominates the RF spectrogram, is similarly retained and folded into the expanded channel dimensions. If left unmitigated, this amplified ambient clutter can severely overwhelm the network’s semantic reasoning.

To decouple the weak signal features from this retained noise, we sequentially integrate the PPA module [21]. This specific pairing is not an arbitrary combination, but a carefully designed architectural synergy. Because the preceding SPD operation physically embeds spatial patterns into the channel depth, the traditional spatial structure is fundamentally altered. Consequently, the resulting tensor strictly requires an advanced mechanism capable of jointly decoding these newly formed cross-channel correlations and the localized spatial dependencies.

Operating directly on the preserved features $X_{spd}$, the PPA module provides a multi-branch parallel architecture perfectly matched to this reorganized representation. It incorporates a cascade of 3×3 convolutions for multi-scale local feature extraction, alongside a 1×1 skip connection ($X_{skip}$) for identity preservation. Crucially, it deploys two Local-Global Attention (LGA) branches operating at distinct patch sizes (2×2 and 4×4). These patch-aware branches are mathematically designed to align with the sub-tensors partitioned by the SPDConv. This precise alignment allows the network to meticulously evaluate local patch dependencies while establishing a global receptive field. Let $Conv_{3\times 3}^{\left(i\right)}$ denote the *i*-th independent convolutional layer in the cascade, where each layer maintains its own distinct set of learnable parameters without weight sharing. The intermediate fused tensor $X_{fuse}$ is then aggregated via element-wise addition:

(1) $$X_{fuse}=\sum_{i=1}^{3}{\mathrm{Conv}}_{3\times 3}^{\left(i\right)}\left(X_{spd}\right)+{\mathrm{LGA}}_{2}\left(X_{skip}\right)+{\mathrm{LGA}}_{4}\left(X_{skip}\right)+X_{skip}.$$

To further distill this enriched feature representation, $X_{fuse}$ passes through a dual-attention mechanism. It initially enters an Efficient Channel Attention (ECA) module [22]. Unlike traditional channel attention, ECA directly recalibrates the expanded channel weights—which now encode rich spatial footprints—without dimensionality reduction. This is immediately succeeded by a Spatial Attention (SA) module, exploiting max-pooling and average-pooling across the channel axis to generate a robust spatial mask that represses irrelevant ambient clutter. The final output is formulated as:

(2) $$F_{out}=\mathrm{SA}\left(\mathrm{ECA}\left(X_{fuse}\right)\right).$$

Ultimately, the synergy between SPDConv and PPA establishes an integrated feature refinement mechanism. SPDConv secures detection sensitivity by ensuring microscopic UAV pixels survive the decimation process, whereas PPA provides the necessary precision by explicitly decoding the folded patch dependencies and stripping away co-located noise. This complementary mechanism empowers the SPA-block to act as a highly selective filter, isolating transient signals in extreme noise-dominant environments.

### 3.3. ATFL

While the SPA block successfully extracts features for microscopic targets, architectural improvements alone cannot resolve the optimization bottleneck in RF spectrograms. Even with spatial preservation, background noise still dominates the image tensor. During backpropagation, the small gradients generated by rare frequency-hopping signals are often overwhelmed by the large gradients accumulated from easily classified background pixels. Due to this extreme foreground–background imbalance, standard Binary Cross-Entropy (BCE) loss and conventional Focal Loss with a static focusing parameter are inadequate [23,24,25].

To address this optimization gap, we introduce the ATFL. ATFL replaces static weight penalization with a batch-aware, dynamic modulation factor $M(p_{t})$ to adjust gradient flows based on the network’s training status [26]. Let $p_{t}$ denote the estimated probability for the ground-truth class. To assess the overall learning difficulty of the current training batch, ATFL calculates the exponential moving average (EMA) of the batch’s mean prediction probability, denoted as ${\hat{p}}_{c}$. It then dynamically derives the focusing parameter as $\gamma =-\ln({\hat{p}}_{c})$. This adaptive mechanism allows the model to adjust its penalization strength in response to fluctuating signal-to-noise ratios across different batches [27].

To complement the filtered features from the SPA block, ATFL applies a piecewise asymmetric modulation strategy pivoting on a confidence threshold of 0.5. For easily classified, high-confidence samples (typically the background noise suppressed by the PPA module, where $p_{t}>0.5$), the modulation factor attenuates their gradient contributions using the dynamic γ. Conversely, for hard-to-detect samples (the microscopic UAV signals preserved by SPDConv, where $p_{t}\leq 0.5$), the mechanism introduces a regulatory hyperparameter λ (set to 1.5 in our experiments) to elevate the compensation baseline to $\lambda -p_{t}$. It also applies a dynamic exponent of $-\ln(p_{t})$. This piecewise modulation factor $M(p_{t})$ is defined as:

(3) $$M\left(p_{t}\right)=\left\{\begin{array}{cc} {\left(1-p_{t}\right)}^{\gamma }, & \mathrm{if}\;p_{t}>0.5\\ {\left(\lambda -p_{t}\right)}^{-\ln(p_{t})}, & \mathrm{if}\;p_{t}\leq 0.5 \end{array}\right..$$

From a mathematical perspective, the exponent $-\ln(p_{t})$ in the lower branch theoretically diverges towards infinity as $p_{t}\to 0$. To guarantee numerical stability during the actual forward and backward passes, $p_{t}$ is strictly clamped with a small lower-bound epsilon $\left(\epsilon =10^{-6}\right)$ before the logarithmic operation. Furthermore, our training pipeline inherently employs gradient clipping with a maximum gradient norm set to 0.1 to prevent any anomalous gradient explosions caused by extreme sample predictions, ensuring stable convergence.

The final ATFL objective incorporates this modulation factor directly into the standard BCE formulation:

(4) $$L_{ATFL}=M\left(p_{t}\right)\cdot L_{BCE}.$$

Ultimately, ATFL and the SPA block form a complete detection ecosystem. During the forward pass, the SPA block isolates the physical footprints of transient signals. During the backward pass, ATFL amplifies the corresponding gradients, ensuring that these weak features receive sufficient weight updates [28]. By combining spatial feature preservation with mathematical gradient scaling, this approach significantly reduces missed detections without adding computational overhead during inference.

## 4. Experiments

### 4.1. Datasets and Evaluation Metrics

To validate the proposed SPA-DETR architecture, we conduct experiments on a custom object detection dataset [29]. This dataset is entirely generated by converting the raw In-phase and Quadrature (I/Q) frequency data from the RFUAV benchmark [30] into 2D time–frequency spectrograms. Unlike simulated collections, the original RFUAV benchmark stands as a highly comprehensive dataset in this domain. Specifically, it comprises approximately 1.3 TB of raw 1D I/Q streams collected from 37 distinct UAV models using Universal Software Radio Peripheral (USRP) devices in complex real-world environments. To adapt this massive raw data for our visual detection framework, we transform it into 2D time–frequency spectrograms using the Short-Time Fourier Transform (STFT) [31]. This preprocessing step visualizes the energy distribution of the transient pulses, forming the direct visual input for our network.

We partition the dataset into 3340 training samples, 946 validation samples, and 477 testing samples. For quantitative evaluation, we follow the standard MS COCO protocol [32]. The primary performance metrics are the mean Average Precision (mAP) computed over multiple Intersection over Union (IoU) thresholds, specifically $mAP_{50}$ and the more stringent $mAP_{50:95}$. Because our core focus is capturing microscopic frequency-hopping footprints, we include the Average Precision for small objects ($AP_{S}$) to specifically measure detection sensitivity for tiny targets. Finally, to assess the model’s viability for deployment on constrained edge devices, we report the parameter count (Params), floating-point operations (FLOPs), and real-time inference speed (FPS).

### 4.2. Implementation Details

We implement the detection pipeline using PyTorch (v2.0.0) [33] and train all models on a single NVIDIA GeForce RTX 4090 GPU (NVIDIA, Santa Clara, CA, USA) with 24GB of VRAM. To balance computational cost and feature resolution, we resize all input spectrograms to 640×640 pixels before training. We train SPA-DETR and all baseline models to full convergence. Specifically, we train the proposed SPA-DETR for 300 epochs with a batch size of 16. We optimize our architecture end-to-end using the AdamW optimizer [34], setting the initial learning rate to $1\times 10^{-4}$ and the weight decay to $1\times 10^{-4}$. To ensure early training stability, we apply a warmup schedule for the first 3 epochs. For a fair comparison, we train the benchmark models (e.g., the YOLO series [35,36]) according to their officially recommended hyperparameters and training schedules.

For data augmentation, we apply a domain-specific strategy tailored for time–frequency representations. Unlike natural image processing, we exclude spatial-destructive techniques such as Mosaic [37] and Mixup [38] to preserve the physical structure and coherence of the UAV RF pulses. Instead, we apply controlled geometric transformations, including translation and scaling, to simulate physical time delays and multipath fading. We also use random erasing [39] to mimic burst channel interference and localized occlusions. This domain-aware augmentation strategy ensures a realistic evaluation for all subsequent benchmark comparisons and ablation studies.

To ensure a fair and reproducible assessment of inference speed, all FPS measurements reported in this study were conducted under identical hardware and software conditions. Specifically, we measured the inference throughput on a single RTX 4090 GPU using a fixed batch size of 16. All models, including the comparative baselines, were executed using their native PyTorch weights (.pt) in standard evaluation mode with Convolution Batch Normalization (Conv-BN) fusion applied. We explicitly excluded external inference engines or hardware-specific optimizations, such as TensorRT or TorchScript, to reflect the architectural efficiency natively. For each model, the final FPS was calculated by averaging the execution latency over 1000 test iterations, immediately following a 200-iteration GPU warm-up phase.

### 4.3. Ablation Study

To evaluate the individual contributions and interactions of our proposed modules, we conduct ablation experiments on the RFUAV dataset (Table 1). The baseline RT-DETR-R18 struggles to locate transient frequency-hopping signals due to their small physical footprints, yielding an $mAP_{50:95}$ of 81.9% and an $AP_{S}$ of 80.4%.

Analyzing the structural modifications reveals that adding modules in isolation provides steady gains. As indicated in Table 1, integrating SPDConv alone improves the $mAP_{50:95}$ to 83.2% (a 1.3% increase). While it retains more spatial pixels, the simultaneous retention of background noise limits the overall accuracy. Similarly, deploying only the PPA module yields an $mAP_{50:95}$ of 84.2%. However, combining both modules into the complete SPA block produces a strong synergistic effect, increasing the $mAP_{50:95}$ to 85.7% and the small-object $AP_{S}$ to 84.3%. This performance growth effectively validates our theoretical inference in Section 3.2: the spatial feature reorganization and attention decoding mechanisms act as a synergistic signal isolation framework.

Beyond structural changes, modifying the optimization function addresses the optimization bottleneck. Replacing the standard BCE loss with ATFL alone improves the baseline’s $mAP_{50:95}$ to 82.8% (a 0.9% increase). While the isolated gain is modest, ATFL proves its value when integrated into the full system. Importantly, this optimization occurs entirely during the backward pass, improving performance without adding network parameters or increasing inference computational cost (remaining at 19.90 M and 57.2 GFLOPs for the baseline version).

Furthermore, regarding the regulatory hyperparameter λ in ATFL, its general sensitivity and threshold stability have been systematically investigated in its foundational literature. Following a similar evaluation paradigm, we specifically explored its behavior across the extreme imbalance regimes unique to RF spectrograms. While the original literature suggests higher values (e.g., λ=3.5) for certain infrared imagery, empirical observations on our RFUAV dataset indicate that setting λ too high (λ≥2.0) over-amplifies the loss for uncertain background noise, causing training instability and degrading convergence. Conversely, a lower setting (λ=1.0) fails to provide sufficient gradient compensation for weak signals. The selected setting of λ=1.5 acts as an optimal threshold tailored for RF characteristics, dynamically maintaining training stability while ensuring high sensitivity to microscopic transient targets.

When integrating both the SPA block and ATFL, the complete SPA-DETR architecture achieves 86.8% in $mAP_{50:95}$ and 85.6% in $AP_{S}$. Compared to the native RT-DETR-R18 baseline, this represents a substantial absolute improvement of 4.9% and 5.2% in $mAP_{50:95}$ and $AP_{S}$, respectively. Simultaneously, the model maintains a real-time inference speed of 235.2 FPS, confirming that the proposed approach successfully balances high-precision detection with the practical constraints of real-world deployment [40].

Consistent with our rigorous evaluation protocol, all ablation results reported in Table 1 represent the average outcomes of strictly three independent runs (3×) using different random seeds. To explicitly confirm the statistical significance of our architectural enhancements, we detail the standard deviations for the primary $mAP_{50:95}$ metric across different configurations: Baseline (±0.16%), +SPDConv (±0.17%), +PPA (±0.14%), +SPA-Block (±0.15%), and +ATFL (±0.18%). As demonstrated, the progressive performance gains achieved by each proposed module (e.g., an absolute +1.3% from SPDConv and an additional +1.5% from the integrated SPA-Block) significantly exceed these marginal fluctuations. This confirms that the effectiveness of our proposed components is statistically robust and not an artifact of random seed selection.

### 4.4. Comparison with State-of-the-Art

To evaluate the detection performance of SPA-DETR, we benchmark it against mainstream object detectors on the RFUAV dataset. The comparative models include the widely used YOLO series (YOLOv8s, YOLOv8m [41], and YOLOv10m), the native RT-DETR-R50 equipped with a deeper backbone, and a recent specialized small-object variant, EABI-DETRv2 [42]. Table 2 summarizes the quantitative results.

Analyzing the quantitative results reveals that SPA-DETR achieves consistent improvements across all accuracy metrics. Compared to YOLOv8m, which obtains high accuracy among the comparative baselines (yielding an $mAP_{50:95}$ of 85.5%), our model achieves a superior performance of 86.8%. For the small-object-specific $AP_{S}$, SPA-DETR outperforms all comparative models with a score of 85.6%. These results confirm that the synergistic design, combining spatial feature preservation with asymmetric gradient scaling, effectively handles microscopic RF footprints.

It is worth noting that all evaluated models achieve a near-saturated $mAP_{50}$ (exceeding 95%). This phenomenon is intrinsically tied to the visual characteristics of RF spectrograms. Unlike natural images with complex semantic textures, RF signals appear as distinct high-energy discrete regions with relatively simple morphologies. Consequently, achieving a loose localization (IoU ≥ 0.5) is a straightforward task for modern deep learning detectors. However, the true challenge in practical electronic countermeasures is not merely finding the general location of a signal, but precisely delineating the boundaries of microscopic, transient pulses amid severe ambient noise. This difficulty is accurately reflected in the more stringent $mAP_{50:95}$ and $AP_{S}$ metrics, where models demonstrate significant performance divergence. This observation explicitly justifies our architectural focus on optimizing for these stricter criteria rather than the saturated $mAP_{50}$.

Furthermore, the comparative experiments highlight a critical observation regarding model scaling and efficiency. When expanding the native RT-DETR from a ResNet-18 to a ResNet-50 backbone, the parameter count more than doubles (from 19.90 M to 41.97 M), and the $mAP_{50:95}$ increases to 86.0%. Although the deeper backbone provides a 4.1% gain over the R18 baseline, the proposed SPA-DETR (86.8%) still surpasses this much heavier RT-DETR-R50 by 0.8% in $mAP_{50:95}$ using only 23.74 M parameters. This comparison demonstrates that targeted architectural interventions, such as the SPA block, are significantly more parameter-efficient than conventional model scaling for decoding time–frequency targets.

Beyond standard baselines, to comprehensively evaluate our method against specialized architectures, we included EABI-DETRv2, a recent state-of-the-art RT-DETR variant explicitly designed for detecting microscopic aerial objects. It incorporates efficient multi-scale attention, bidirectional feature fusion, and the Focaler-MPDIoU loss function to optimize hard sample localization. As shown in Table 2, despite these specialized structural optimizations and advanced regression loss, our proposed SPA-DETR consistently achieves superior performance. For instance, while EABI-DETRv2 yields an impressive $mAP_{50:95}$ of 84.7% and an $AP_{S}$ of 83.3%, SPA-DETR maintains a distinct advantage. This empirically demonstrates that the integration of the SPA block and our proposed ATFL provides a more robust and accurate solution for microscopic target detection in complex electromagnetic environments than existing domain-specific alternatives.

It is worth noting that all performance metrics reported in Table 2 represent the average outcomes of strictly three independent runs (3×) using different random seeds. To provide a comprehensive variance analysis and avoid point-estimate reporting, we detail the standard deviations for the primary $mAP_{50:95}$ metric across all comparative baselines: YOLOv8s (±0.14%), YOLOv8m (±0.17%), YOLOv10m (±0.22%), RT-DETR-R18 (±0.16%), EABI-DETRv2 (±0.19%), and the strongest baseline RT-DETR-R50 (±0.18%). In comparison, our proposed SPA-DETR exhibits a variance of ±0.15%. Crucially, our absolute improvement margins significantly exceed these fluctuations. For instance, the 0.8% absolute margin over RT-DETR-R50 is substantially larger than its ±0.18% variance. This confirms that our broader superiority over all evaluated models is statistically robust and originates directly from our architectural enhancements rather than experimental variance.

Finally, while substantially improving detection accuracy, SPA-DETR maintains a practical balance between computational complexity and inference speed. Its computational requirement of 71.1 GFLOPs is lower than that of YOLOv8m (78.7 GFLOPs). Although the lightweight YOLO variants operate at higher absolute frame rates (e.g., YOLOv10m at 400.4 FPS), the 235.2 FPS throughput of SPA-DETR far exceeds the real-time processing threshold required by practical electronic countermeasure systems. Overall, SPA-DETR establishes a highly favorable trade-off between speed, parameter efficiency, and accuracy for microscopic signal detection.

### 4.5. Qualitative Evaluation and Visualization

**Figure 3:**
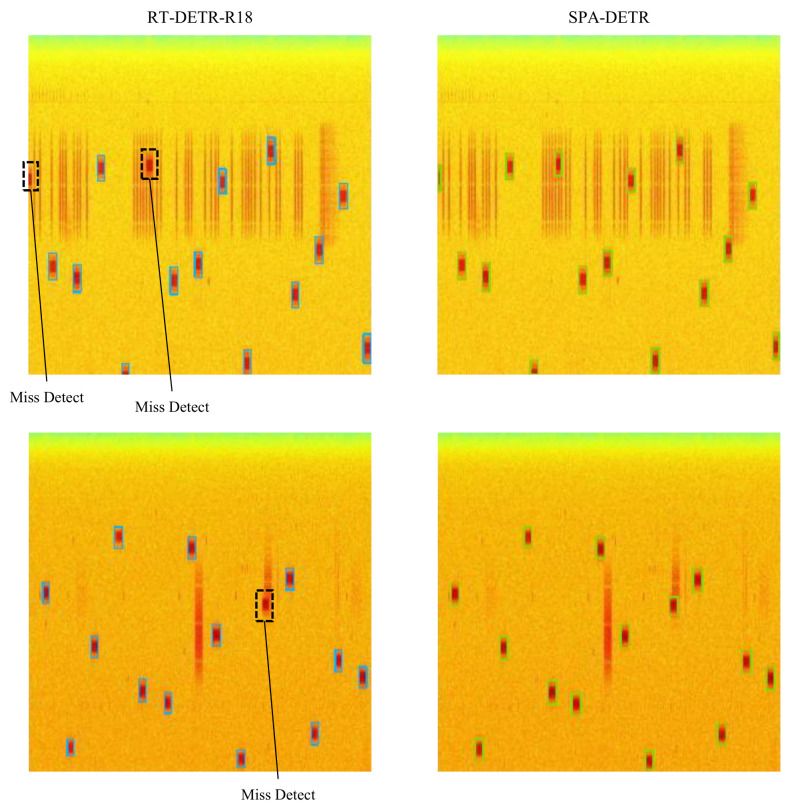

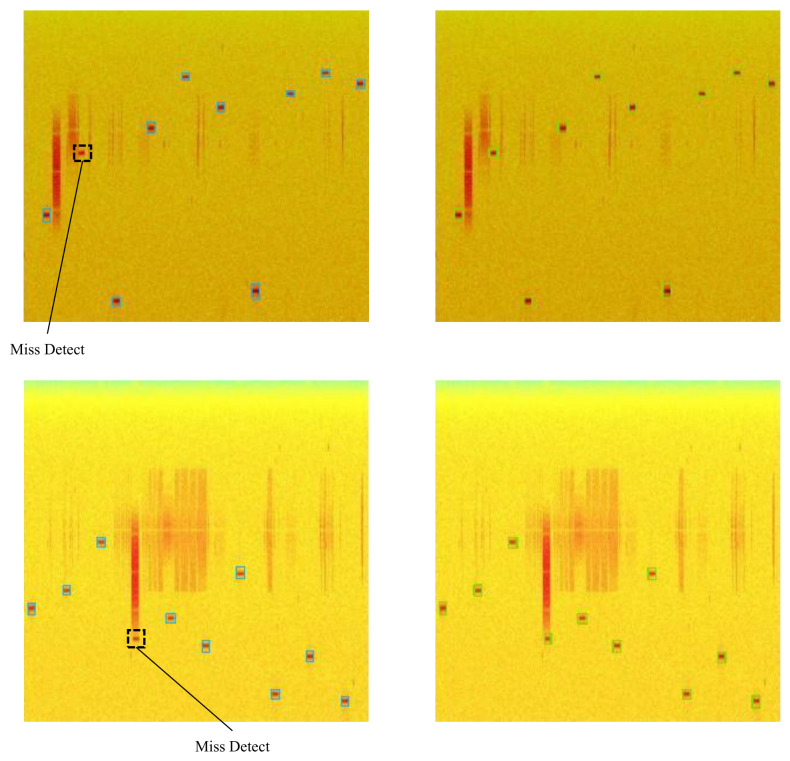
Qualitative detection results on the RFUAV dataset. The left column shows the detection results of the baseline RT-DETR-R18, where dashed boxes indicate missed detections (false negatives). The right column demonstrates the successful localizations achieved by the proposed SPA-DETR in the identical spectrograms.

To provide an intuitive understanding of the practical detection performance, Figure 3 presents a qualitative comparison between the baseline RT-DETR-R18 and the proposed SPA-DETR. We specifically select RT-DETR-R18 to serve as the native baseline, aiming to visually demonstrate the direct architectural improvements brought by our method. The selected test samples primarily contain microscopic frequency-hopping signals embedded within complex background noise, representing the most challenging scenarios in practical electronic countermeasures.

Visual inspection of these scenarios reveals a significant difference in detection reliability. As highlighted in the left column of Figure 3, RT-DETR-R18 is prone to missed detections (false negatives) when encountering transient frequency-hopping pulses with minimal physical footprints. This phenomenon directly reflects the spatial information loss typical of traditional convolutional networks during deep feature extraction [43]. In contrast, the right column demonstrates that SPA-DETR consistently captures these extremely small targets. This visual evidence corroborates the effectiveness of the SPA block in preserving spatial details and isolating weak signals from severe ambient interference.

The improved performance can be attributed to the synergy between architectural preservation and optimization. While standard detectors often struggle to distinguish microscopic signals from background clutter, SPA-DETR establishes a clear separation between the target and noise. This is consistent with our quantitative findings, proving that the integration of the SPA block and ATFL effectively enhances the network’s sensitivity to transient, low-energy targets. These visualization results further confirm the robustness of the SPA-DETR architecture when processing complex time–frequency features in real-world environments.

## 5. Conclusions

This study presents SPA-DETR, a custom object detection architecture designed to localize transient UAV signals within complex RF spectrograms. To overcome the spatial information loss inherent in standard convolutional downsampling, we introduce the SPA block. By replacing conventional pooling operations across the backbone and multi-scale neck, the SPA block maintains global spatial fidelity throughout the feature hierarchy. Integrating SPDConv with a PPA module, this design establishes a closed-loop feature purification system. It successfully preserves the microscopic footprints of frequency-hopping pulses while filtering out ambient background noise.

Furthermore, to address the optimization bottleneck caused by severe foreground–background imbalance, we propose the ATFL. Operating entirely during backpropagation, ATFL mitigates gradient domination through asymmetric gradient scaling. This mechanism directs the network’s focus toward hard-to-detect microscopic targets without adding computational overhead during inference. Extensive evaluations on the RFUAV dataset validate these contributions. SPA-DETR achieves an $mAP_{50:95}$ of 86.8% and an $AP_{S}$ of 85.6%, improving upon the baseline RT-DETR-R18 by 4.9% and 5.2%, respectively. It substantially outperforms mainstream models like RT-DETR-R50 and YOLOv8m while maintaining a real-time inference speed of 235.2 FPS. Ultimately, this research demonstrates that targeted architectural modifications guided by underlying signal physics are significantly more effective for processing time–frequency targets than conventional model scaling. Future work will focus on the quantized deployment of this architecture on resource-constrained edge devices [44] and evaluate its generalizability across a wider range of electromagnetic signal morphologies [45].

## Figures and Tables

**Table 1:** Ablation study of the proposed components on the RFUAV dataset. The SPA block integrates both SPDConv and PPA.

Model	SPDConv	PPA	ATFL	mAP_50:95_	mAP_50_	AP*_S_*	Params (M)	GFLOPs	FPS
Baseline (RT-DETR-R18)				81.9	99.7	80.4	19.90	57.2	235.6
+SPDConv	✓			83.2	99.7	81.7	19.26	56.8	340.0
+PPA		✓		84.2	99.7	82.7	24.38	71.6	265.5
+SPA-Block (SPD+PPA)	✓	✓		85.7	99.8	84.3	23.74	71.1	235.9
+ATFL (on Baseline)			✓	82.8	99.7	81.4	19.90	57.2	234.8
**Ours (SPA-DETR) **	✓	✓	✓	**86.8**	**99.8**	**85.6**	**23.74**	**71.1**	**235.2**

**Table 2:** Quantitative comparison with representative object detectors on the RFUAV dataset.

Model	mAP_50:95_	mAP_50_	AP*_S_*	Params (M)	GFLOPs	FPS
YOLOv8s	82.8	95.3	82.0	11.13	28.5	997.2
YOLOv8m	85.5	95.4	84.7	25.85	78.7	421.5
YOLOv10m	80.6	95.9	79.0	16.47	63.5	400.4
RT-DETR-R18	81.9	99.7	80.4	19.90	57.2	235.6
RT-DETR-R50	86.0	99.8	84.8	41.97	125.7	158.3
EABI-DETRv2	84.7	99.7	83.3	13.56	85.4	224.6
**Ours (SPA-DETR)**	**86.8**	**99.8**	**85.6**	**23.74**	**71.1**	**235.2**

## Data Availability

The raw RFUAV I/Q data is available at https://github.com/kitoweeknd/RFUAV/ (accessed on 8 November 2025), reference [30]. The processed time–frequency spectrogram dataset used in this study is openly available at https://universe.roboflow.com/mmdrone-fj6em/final_2-qqpzd (accessed on 20 January 2026), reference [29].
